# Long-term follow-up of non-operated patients with symptomatic gallbladder stones: a retrospective study evaluating the role of Hepatobiliary scanning

**DOI:** 10.1186/s12876-015-0368-1

**Published:** 2015-10-15

**Authors:** Keun Soo Ahn, Ho-Seong Han, Jai Young Cho, Yoo-Seok Yoon, Chulhan Kim, Won Woo Lee

**Affiliations:** 1 Department of Surgery, Keimyung University Dongsan Medical Center, 56 Chungho-ro, Jung-gu, Daegu city,, 41931 Korea; 2 Department of Surgery, Seoul National University Bundang Hospital, Seoul National University College of Medicine, Korea, 300 Gumi-dong, Bundagn-gu, Seongnam city, Gyeonggido 463-707 Korea; 3 Department of Nuclear Medicine, Korea University Ansan Hospital, Ansan city, Gyeonggido, 425-707 Korea; 4 Department of Nuclear Medicine, Seoul National University Bundang Hospital, Seoul National University College of Medicine, Korea, Gyeonggido, 463-707 Korea

**Keywords:** Gallbladder ejection fraction, Gallstone, Hepatobiliary scan

## Abstract

**Background:**

To assess hepatobiliary (HB) scans for predicting recurrent symptoms in nonoperated patients with mild or vague symptomatic gallstones.

**Methods:**

Data of 170 patients with symptomatic gallstone and who had not undergone cholecystectomy were retrospectively enrolled. These patients were divided into two groups according to whether or not operations were performed due to recurrent symptoms during the follow-up period. The demographic factors and gallbladder ejection fraction (GBEF) of HB scans were compared between the groups. Additionally, symptom-free rate was obtained beginning from the date of the HB scan to the date of surgery, and analyzed based on the level of GBEF.

**Results:**

Among the 170 enrolled patients, two patients who underwent cholecystectomy for other disease were excluded. Thirty-four patients underwent cholecystectomy due to recurrent symptoms (OP group), and the remaining 136 patients did not experience recurrent symptoms and therefore did not undergo cholecystectomy (non-OP group). In the OP group, the mean GBEF was significantly lower than that of the non-OP group (28.8 ± 29.9 *vs.* 66.3 ± 20.0; *P* < 0.001). The rate of lower GBEF (<30 %, including non-visualization of the gallbladder) was significantly higher in the OP group than the non-OP group (54.9 *vs.* 5.1 %; *P* < 0.001). In patients with non-visualization of the gallbladder or GBEF <30 %, the 10-year symptom-free rate was significantly lower than those with a GBEF ≥ 30 % (19.8 % *vs.* 81.9 %; *P* < 0.001).

**Conclusion:**

HB scanning is a useful objective modality to differentiate gallstone-related symptoms from other etiologies and predict recurrent symptoms.

## Background

Most cases of gallstone disease are asymptomatic [1, 2] and incidentally found during routine abdominal ultrasonography [3]. The average risk of developing symptoms in gallstone disease is 2.0–2.6 % per year [4]. When symptoms do occur, treatment by cholecystectomy is advised [5–8]. Thus, the diagnosis of “symptomatic” gallbladder (GB) stones is an essential step in the decision to operate. A symptom of gallstone disease is biliary colic, which is a severe steady pain lasting more than 15–30 min, usually located in the epigastrium and/or right upper quadrant, and sometimes radiating to the back [9]. However, if patients have nonspecific symptoms, such as indigestion, discomfort, fat intolerance, and bloating in the presence of a GB stone, it is difficult to determine if these symptoms are associated with GB disease. Gallstone-associated symptoms may occasionally be vague, and are usually confused with other symptoms of gastrointestinal disease. This may explain why biliary symptoms are not relieved in 6–33 % of patients after cholecystectomy [10–14]. Cholecystectomy in such patients is delayed because of the vague nature of symptoms, and a significant portion of these patients do not undergo the procedure even though they have symptoms [15].

There has been no useful objective modality to differentiate gallstone-related symptoms from other etiologies or to predict symptom recurrence. Hepatobiliary (HB) scanning is a sensitive diagnostic modality for acute cholecystitis [16–20], and is also useful for predicting the severity of cholecystitis [21]. However, the associations between HB scan results and gallstone-related symptoms have not been well elucidated [22, 23]. The aim of this retrospective study was to assess the role of HB scanning for predicting symptom recurrence in patients with vague symptomatic gallstone disease who have not undergone cholecystectomy.

## Methods

### Study population and study design

At our institution, HB scanning is routinely performed for symptomatic GB stones, except for patients who require urgent treatment due to acute cholecystitis. From January 2004 to December 2009, HB scanning was performed in 2274 patients with symptomatic gallstones. Cholecystectomy was performed in 2058 patients. There were 216 patients with symptomatic GB stones that did not undergo the operation primarily due to vague and nonspecific symptoms, among which 46 patients could not be followed-up. The remaining 170 patients were enrolled in this study. Follow-up of these patients was performed via outpatient clinic or telephone interviews by third party non-physicians, and the last follow-up was conducted in May 2014. The symptom-free rate was obtained based on the date the HB scan was performed to the date of surgery due to recurrent symptom, or the end of the study in May 2014. This was a retrospective study based on prospectively recorded registry. This retrospective study protocol was reviewed and approved by the institutional review board at Seoul National University Bundang Hospital (B:1010/113–102). The consents from patients for this retrospective study were waived by the institutional review board at Seoul National University Bundang Hostpial (B:1010/113–102).

### HB scanning

The HB scan protocol of our hospital was described previously [21]. Following an overnight fast, HB scintigraphy was performed using ^99m^Tc-mebrofenin with a bolus intravenous infusion of 15 mCi. If no radioactivity was detected in the GB area at 4 h after the infusion, the patient was classified as having non-visualization of the GB, and the scintigraphic recording was finished. For the patients with detectable radioactivity that corresponded to the GB area, the infusion of the radioactive marker and the scintigraphic recordings were continued. The patient then ingested a standard fat meal consisting of an omelet and a glass of water, and scintigraphic recordings were performed every 30 s for 60 min. By generating a region of interest over the GB area, the maximum GB ejection fraction (GBEF) was calculated.

### Statistics

All analyses were performed using SPSS 17.0 for Windows (SPSS Inc., Chicago, IL, USA). The continuous parameters in each group were compared by the Mann–Whitney *U* test, and the categorical parameters were compared using the *χ*^*2*^ test or Fisher’s exact test. Recurrent symptom-free rate was calculated using the Kaplan–Meier method. The continuous, normally distributed data are presented as mean ± standard deviation, and the nonparametric values are expressed as median (range). Differences were considered significant at *P* < 0.05.

## Results

### Demographic data and clinical outcome during follow-up

The patient population was comprised of 77 men and 93 women with a mean age of 54.3 y. Initially, 29.4 % (50/170) of patients complained of biliary colic and 70.6 % (120/170) had upper abdominal discomfort, indigestion or intolerance of a fatty meal without biliary colic. Thirty-four of the patients underwent cholecystectomy during a mean follow-up period of 58.5 ± 29.3 mo (range: 4.0–124.0 mo). Thirty-two (32/170; 18.8 %) patients experienced recurrent symptoms or symptoms of acute cholecystitis and underwent cholecystectomy (OP group); cholecystectomy was performed on two patients without any recurrent symptoms for other unrelated diseases and they were excluded from the analysis. A total of 136 patients did not experience any recurrent symptoms and did not undergo cholecystectomy (non-OP group). There were no significant differences in sex, age, or initial symptoms between the groups (Table 1). For the patients in the OP group, 29 were followed-up over a median of 24 mo (range: 4.0–75.0 mo) after cholecystectomy; 96.6 % (28/29) of the patients showed disappearance of symptoms, but one patient complained of sustained symptoms.

**Table 1:** Patient characteristics

Variable	OP group (*n* = 32)	Non-OP group (*n* = 136)	*P*
Age, y^c^	52.2 ± 14.8	54.7 ± 14.1	0.391^a^
Sex, *n*^d^	19/13	57/79	0.056^b^
Presence of underlying disease, *n* (%)	4 (12.5)	28 (20.6)	0.216^b^
Initial symptom, *n* (%)			0.591^b^
Biliary colic	12 (37.5)	38 (27.9)	
Epigastric discomfort	18 (56.3)	84 (61.9)	
Indigestion	1 (3.1)	11 (8.2)	
Fat intolerance	1 (3.1)	3 (2.2)	

Abbreviations: *OP* Cholecystectomy due to recurrent symptoms during follow-up period

^a^Mann–Whitney *U* test

^b^χ^*2*^ test

^c^Data is presented as mean ± standard deviation

^d^Data is presented a (male/female)

### HB scan results and symptom recurrence

**Fig. 1:**
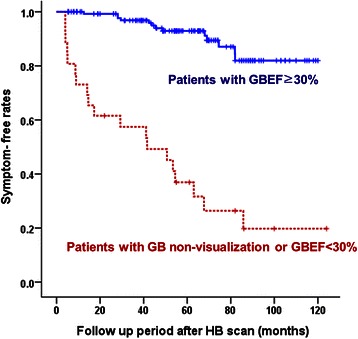
Symptom-free rates. GB, gallbladder; GBEF, gallbladder ejection fraction; HB, hepatobiliary

The mean GBEF was significantly lower in the OP group that in the non-OP group (*P* < 0.001) (Table 2). Moreover, the GBEF values were significantly lower in the OP group when patients were classified according to symptoms (all *P* < 0.05). The GBEF was < 30 % for significantly more patients in the OP group compared to the non-OP group (*P* < 0.01), including 12 patients with non-visualization of the GB in the OP group and two patients in the non-OP group. In patients with GBEF < 30 %, the 10-year symptom-free rate was significantly lower than those with a GBEF ≥ 30 % (19.8 *vs.* 81.9 %; *P* < 0.001) (Fig. 1).

**Table 2:** Hepatobiliary scan results

Result	OP group (*n* = 32)	Non-OP group (*n* = 136)	*P*
Mean GBEF, %^c^	28.8 ± 29.9	66.3 ± 20.0	<0.001^a^
With biliary colic	18.2 ± 30.1 (*n* = 12)	61.5 ± 22.0 (*n* = 38)	0.001^a^
With epigastric discomfort	41.3 ± 33.5 (*n* = 18)	65.8 ± 20.5 (*n* = 84)	0.011
With indigestion and fat intolerance	35.2 ± 1.4 *(n =* 2)	72.3 ± 17.3 (*n* = 14)	<0.001^a^
GBEF < 30 % or nonvisualization of GB, *n* (%)	19 (59.4)	7 (5.1)	<0.001^b^

Abbreviations: *GBEF* Gallbladder ejection fraction, *OP* cholecystectomy due to recurrent symptoms during follow-up period

^a^Mann–Whitney *U* test

^b^χ^*2*^ test

^c^Data is presented as mean ± standard deviation

## Discussion

If the GB-associated symptoms are distinct or severe, the decision to operate is straightforward. Therefore, most of the patients with symptomatic GB stones will undergo cholecystectomy. However, there is a significant portion of patients with symptoms that do not undergo operation, such as those with vague symptoms, for whom observation is initially preferred.

For patients with gallstone-related biliary colic, the symptom relief rate after cholecystectomy is higher than that for patients with other nonspecific symptoms [12, 24]. However, additional symptoms in some patients can be vague, and surgery is not usually performed. In the present study, 80 % of patients who described their symptoms as “biliary colic”, though mild, did not develop recurrent symptoms or undergo subsequent cholecystectomy. Furthermore, symptom recurrence did not differ in patients with other nonspecific symptoms, such as epigastric discomfort, indigestion and fat intolerance. These findings demonstrate that mild or vague subjective symptoms, including patient-reported “biliary colic”, are not accurate indicators for differentiating specific gallstone-associated symptoms from those of another etiology.

The pain associated with symptomatic GB stones is related to decreased gallbladder motor function that is secondary to an intermittent and partial obstruction of the cystic duct or functionally impaired GB [25], which can be revealed by HB scanning without interobserver variability. A normal finding on an HB scan indicates normal patency of the cystic duct, and patients with gallstones that are not causing symptoms typically have normal gallbladder contractility. The HB scan may be useful for predicting the clinical course of symptomatic GB stones and to select patients for cholecystectomy, such as those with biliary colic showing low GBEF [26–30]. In the present study, patients who experienced recurrent symptoms had lower GBEF values and rate of GB non-visualization, and the 10-year symptom-free rate was significantly lower in patients with GBEF < 30 % compared to those with a GBEF ≥ 30 % (19.8 *vs.* 81.9 %; *P* < 0.001). According to this result, when a patient has a nonspecific symptom such as indigestion or discomfort, cholecystectomy should be considered if an HB scan reveals low GBEF. In contrast, when GBEF is ≥ 30 % in patients with mild and vague symptoms, their symptoms may not be gallstone related. These findings suggest that preoperative HB scanning may be useful to distinguish gallstone-related symptoms from other etiologies, and for the decision to perform cholecystectomy.

Despite the apparent advantages, HB scanning is time consuming for patients who require delayed scans to check for slow emptying, taking several hours to achieve a final diagnosis. Moreover, it requires specialized equipment that may not be available at all institutions. Therefore, HB scanning can be applied to select patients with GB stones who show nonspecific vague symptoms or biliary colic. Further studies are required to confirm if these findings are applicable to patients in countries beyond Asia, such as European or American populations with different dietary habits and compliance to treatment. Additionally, further study with comparison to a control group comprised of those with non-visualization of the GB or GBEF < 30 % and no gallstones is needed to verify the predictive value of HB scanning. Therefore, prospective studies with validated questionnaires and longer follow-up periods are needed.

## Conclusion

HB scanning is a useful, objective modality to differentiate gallstone-related symptoms from other etiologies and to predict symptom recurrence. As such, HB scans can be used to aid in the decision to perform cholecystectomy in patients with vague symptomatic gallstones.

## Abbreviations

HB: Hepatobiliary
GBEF: Gallbladder ejection fraction
OP: Operation
GB: Gallbladder
